# Application of Polysaccharides in Hydrogel Biomaterials

**DOI:** 10.3390/ijms26073387

**Published:** 2025-04-04

**Authors:** Piotr Szatkowski, Zuzanna Flis, Anna Ptak, Edyta Molik

**Affiliations:** 1 Department of Glass Technology and Amorphous Coatings, Faculty of Materials Science and Ceramics, AGH University of Krakow, Al. Mickiewicza 30, 30-059 Krakow, Poland; pszatko@agh.edu.pl; 2 Department of Animal Biotechnology, Faculty of Animal Science, University of Agriculture in Krakow, Al. Mickiewicza 24/28, 31-059 Krakow, Poland; zuzanna.flis@student.urk.pl; 3 Laboratory of Physiology and Toxicology of Reproduction, Institute of Zoology and Biomedical Research, Faculty of Biology, Jagiellonian University, Gronostajowa 9, 30-387 Krakow, Poland; anna.ptak@uj.edu.pl

**Keywords:** hydrogels, biocomposite, natural compounds, wound healing

## Abstract

Natural compounds incorporated into hydrogel materials have been widely used to support wound healing due to their numerous properties. The aim of this research was to produce hydrogel biomaterials with the addition of adjuvants, such as sodium alginate and polyethylene glycol diacrylate (PEGDA) with the addition of ethylene ginger extract (EEI). A thermogravimetric (TG) study, differential scanning calorimetry (DSC), dynamic mechanical analysis (DMA), water absorption testing and microscopic analysis were carried out to determine the properties of the developed dressing. The conducted research showed that the 4%Alg/12%PEGDA hydrogel was characterized by the best water absorption values and the slowest weight loss as a function of temperature. Additionally, the 4%Alg/12%PEGDA hydrogel had the best ability to dissipate stress in its structure. It was found that the addition of the ginger modifier had a negative effect on the water absorption values. Hydrogel containing 4%Alg 12%PEGDA 12%EEI showed the best hydrophilic properties and the highest ionic conductivity. The studies conducted showed that both the addition of PEGDA and EEI to hydrogels affects the increase in acidity of dressings. This is important because maintaining an acidic wound microenvironment is a potential therapeutic strategy for wound management. Therefore, although further research is needed, it is possible that 4%Alg 12%PEGDA 12%EEI hydrogel could be used as a high-performance wound dressing.

## 1. Introduction

Wound healing is a complex biological process that varies in completeness and healing time depending on the type of wound and how much the skin structure has been damaged. An appropriate wound dressing should provide a suitable environment for wound healing and provide a protective barrier between the damaged tissue and external factors. Among the various types of dressings widely used to support wound healing, hydrogels are an excellent choice due to their special properties.

Alginate hydrogels can be produced by various cross-linking methods, and their structural similarity to tissue matrices allows for wide biotherapeutic applications. They are used as wound healing aids and to deliver bioactive agents such as drugs, proteins or natural extracts with health-promoting properties.

Currently, more and more people are interested in alternative and, especially, natural medical solutions that are effective and do not cause side effects. Plant and herbal extracts are increasingly desirable as an alternative therapy in the treatment of many human diseases due to their numerous properties.

Due to its high concentration of bioactive compounds, ginger has a number of health- benefits for the body [1]. Ginger contains active substances such as oleoresin, gingerol, shogaol and flavonoids. Gingerol and shogaol are phenolic components of ginger that have anti-inflammatory, antimicrobial and anticancer effects [2]. Ginger extract can affect the proliferation phase in cut wounds by reducing the number of neutrophils and increasing the number of fibroblasts [3]. Studies by Jamaluddin et al. (2021) have shown that the administration of a 10% ginger extract ointment is very effective in accelerating the healing process of cut wounds [4]. However, in the case of medical ointments and creams, the absorption efficiency can be impaired due to abrasion of the preparation from the wound surface [5]. Therefore, due to the ease of application and use, the integration of plant extracts with dressings may prove to be a breakthrough solution. Gel dressings are characterized by biodegradability, biocompatibility, air permeability and the ability to maintain a physiologically moist wound microenvironment. Additionally, they minimize bacterial infections at the wound site and facilitate their healing [6,7].

The design of a suitable hydrogel dressing enriched with ginger as a bioactive substance of natural origin may demonstrate great therapeutic potential in the wound healing process. Therefore, the aim of the research conducted was to produce hydrogel biomaterials with the addition of supporting substances, such as sodium alginate and polyethylene glycol diacrylate, with the addition of ethylene ginger extract.

## 2. Results

**Figure 1:**
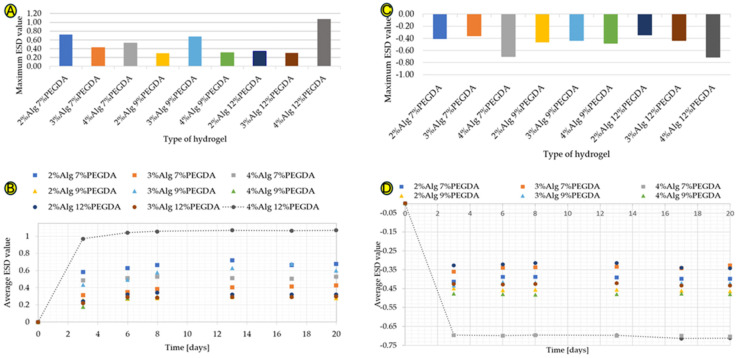
(**A**–**D**) Graphs showing maximum ESD values for hydrogel samples in deionized water (**A**), ESD values of samples immersed in deionized water from time of testing (**B**), the maximum ESD coefficient values for hydrogel samples immersed in ethylene alcohol (**C**), and ESD coefficient values of samples immersed in ethylene alcohol from time of testing (**D**).

The results of the water absorption test in deionized water for hydrogels without the addition of EEI are shown in Figure 1A,B. A significant increase in ESD is observed for each of the hydrogels obtained. However, the highest water absorption is characterized by the hydrogel sample with 4% sodium alginate content and 12% poly(ethylene glycol) diacrylate (4%Alg 12%PEGDA) content. The maximum ESD value is 1.07 (Figure 1A). On the other hand, the lowest water absorption, taking into account the maximum ESD value, which is 0.298, is characterized by the 2%Alg 9%PEGDA hydrogel sample (Figure 1A).

The time-dependent water absorption test (Figure 1B) shows a large increase in the ESD value during the first days of the test. The hydrogel samples absorb a large amount of water, solvation of hydrophilic and hydrophobic groups occurs, and then a plateau phase is visible on the graph. The ESD value increases steadily over the following days of the test, but these are low values from about 0.001 to about 0.070. The amount of water absorbed by a given material depends, among other things, on the hydrophilicity of the substances of which it is composed and on the compressibility of the polymer mesh. This property is extremely important in the context of a wound dressing, which must have a high exudate absorption capacity. The 4%Alg 12%PEGDA hydrogel absorbed the largest amount of water.

The hydrogel samples were also immersed in ethylene alcohol, simulating a hypertonic environment, in order to compare the degree of water loss with the degree of its absorption. The results shown in Figure 1C are different from those shown in Figure 1A. The highest water loss is characteristic of the 4%Alg 12%PEGDA hydrogel, whose minimum ESD result is −0.713, but a very similar ESD value is characteristic of the 4%Alg 7%PEGDA hydrogel. This value is −0.704. The similarity of the water loss for these two samples is visible in Figure 1D.

Reference samples, i.e., hydrogel samples without PEGDA and EEI, are characterized by the highest water absorption values for the hydrogel with 2% sodium alginate content. The ESD test results for these samples are shown in Figure S1a,b (Supplementary Materials). The data obtained in the water absorption and water loss tests for 3%Alg and 4%Alg hydrogels are similar. Comparing the results presented in Figure 1A,B with the results obtained for the reference samples, the addition of PEGDA to the samples with 2% AlgNa content reduces their water absorption degree by 0.168 for 7% PEGDA, by 0.592 for 9% PEGDA, and by 0.548 for 12% PEGDA. For hydrogels with 3% AlgNa content, the lower PEGDA concentrations, 7% and 9%, increase the ESD value (comparing the maximum ESD value) by 0.063 and 0.317, respectively. The 12% PEGDA addition, on the other hand, reduced the water absorption value by 0.06. For the 4%Alg hydrogel, the addition of 7% PEGDA increased the maximum water absorption value by 0.145. The 9% PEGDA addition caused a decrease in this value by 0.073. On the other hand, the 12% addition caused an increase in the ESD value of up to 0.686.

**Figure 2:**
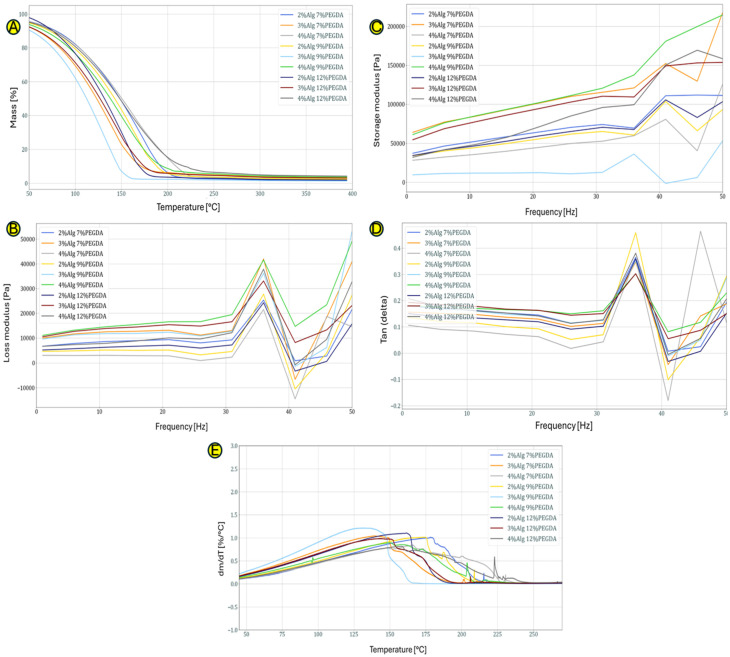
(**A**–**E**) Graphs showing TG curves for the obtained hydrogels without EEI addition (**A**), DTG curves for the obtained hydrogels without the addition of EEI (**B**), the storage modulus versus frequency for hydrogels without EEI addition (**C**), the loss factor as a function of frequency for hydrogels without EEI addition (**D**) and loss modulus versus frequency for hydrogels without EEI addition (**E**).

Figure 2A, which shows the curves obtained as a result of the TG test, shows the percentage loss of mass of the hydrogel samples depending on the temperature. The loss of mass is mainly due to the loss (evaporation) of water. Hydrogels in which the water is permanently bound (the strongest bonds in hydrogels occur when the bonds between polymer chains and water molecules have the highest energy) to the hydrophilic groups of the constituent polymers are characterized by its loss at higher temperatures. The 3% sodium alginate samples lose mass the fastest, probably indicating an unstable bond between the polymer skeleton of the hydrogel and the water molecules. The 4%Alg 12%PEGDA hydrogel loses mass at the slowest rate as a function of temperature, making it a potentially good choice as a material for wounds.

The 4%Alg 7%PEGDA hydrogel sample has similar properties to the 4%Alg 12%PEGDA sample. From the data presented in Table 1, it can be concluded that it loses 50% of its mass at a higher temperature of 153 °C than the 12% PEGDA sample, which loses 50% of its mass at 150 °C. This may indicate better adaptation to the chosen application.

The DTG analysis shown in Figure 2B shows when the mass loss process begins and the intensity with which it occurs. The greater the DTG peak flattening, the slower the hydrogel sample loses water. Due to the successive evaporation of different types of bound water in DTG, we observe a main flattened peak which indirectly defines the nature of the obtained spatial structure of the obtained and tested hydrogel materials.

Figure 2A,B,E depict the curves showing the dependence of the loss modulus on the given test frequency obtained during the DMA test. The value of the loss modulus (Figure 2E) for all hydrogel samples from the frequency of 1 Hz to a frequency equal to 31 Hz is similar and oscillates in the range of 0–2000 Pa. With the increase of the frequency above 31 Hz, we observe fluctuations related to the energy dissipation in the hydrogels. The hydrogel sample with 4% content of sodium alginate and 12% poly(ethylene glycol) diacrylate content is characterized by average loss modulus values compared to the other hydrogels. The jump in the loss modulus around the 35 Hz value means that at this frequency, stress dissipation is more effective, which may indicate that at this frequency, the secondary bonds between the polymer chains forming the hydrogel are broken.

For the storage modulus, similar trends can be observed as in the case of the loss modulus (Figure 2C). Its value up to a frequency of approx. 31 Hz is constant for all hydrogel samples, and with its increase, fluctuations in the storage modulus value are observed. The higher the frequency value at which the prepared hydrogels were tested, the more the material densified, becoming more compact (chains approaching each other with simultaneous evaporation of water from the hydrogel structure). Similarly to the case of the turbidity modulus (Figure 2D), the 4%Alg 12%PEGDA hydrogel is characterized by average values of the storage modulus.

The 2%Alg 7% PEGDA sample differs from the other hydrogel samples, indicating a low level of polymer matrix bonding (e.g., large lengths of the hydrogel main chains and low density of hydrogen bonds) and the weakest spatial cross-linking in this type of hydrogel composite.

**Figure 3:**
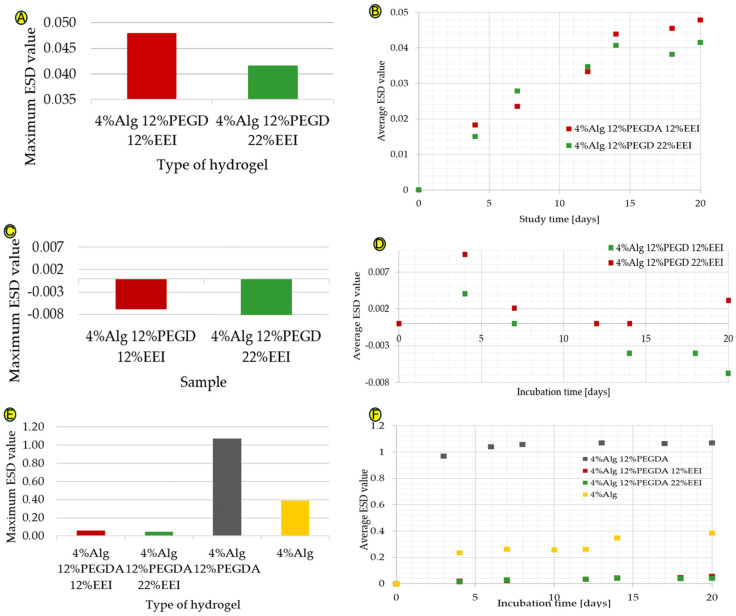
(**A**–**F**) Graphs showing the maximum ESD coefficient values for hydrogel samples with EEI added in deionized water (**A**), the maximum ESD coefficient values versus time for hydrogel samples with EEI added in deionized water (**B**), the maximum ESD coefficient values for hydrogel samples with EEI added in ethylene alcohol (**C**), the maximum ESD coefficient values versus time for hydrogel samples with EEI added in ethylene alcohol (**D**), a comparison of the maximum ESD coefficient values for hydrogel samples without and with EEI additive, in deionized water (**E**), and a comparison of the maximum ESD coefficient values as a function of time for hydrogel samples without and with EEI additive in deionized water (**F**).

The 4%Alg 12%PEGDA hydrogel was selected for modification, as it was characterized by the best absorbency values. This feature is very important in the above-mentioned application, as the dressing must be able to absorb a large amount of wound exudate. Additionally, the hydrogel with the slowest mass loss as a function of temperature was selected, indicating a high degree of binding of its polymer chains with water molecules and was characterized by a relatively high tan(delta) value. Depending on the concentration of the ethylene extract of ginger that was incorporated into the hydrogel structure, various changes in absorbency were observed, as shown in Figure 3A,B. As the amount of EEI increases, an increase in absorbency is observed, which may be due to the formation of additional hydrogen bonds (i.e., an increase in hydrogen bond density). This affects the densification of the polymer network, which makes it difficult for water molecules to penetrate deep into the polymer network. Ginger is hydrophilic; therefore, an increase in the water absorption of the material would be expected. The more hydrophilic groups in the hydrogel structure, the greater its ability to absorb water [8]. The water absorption values are characterized by a minimal increase, the maximum ESD value of the 4%Alg 12%PEGDA 12%EEI hydrogel is 0.048, and for the 4%Alg 12%PEGDA 22%EEI hydrogel, it is 0.042. It is possible that ginger present in the system at low concentrations causes cross-linking of the system, connecting polymer chains with each other, consequently preventing them from binding with water. Creating a system with a higher EEI concentration may have a positive effect on the water absorption values. However, such an approach may involve exceeding the amount of modifiers that may be present in the composite, which often leads to a deterioration in the water absorption properties. This is probably the reason for the low water absorption values of the hydrogel samples with ginger extract—an increase in EEI concentration in the system may have a negative effect on water absorption. The difference in the maximum water absorption value between the hydrogel with 12% EEI content and the hydrogel with 22% EEI content is 0.006.

The results of the hydrogel samples immersed in ethylene alcohol shown in Figure 3C,D are interesting. Alcohol, which simulates a hypertonic environment, should cause water to flow out of the hydrogel samples; however, during the first test there is an increase in the ESD value, suggesting that the hydrogels have absorbed some alcohol. The alcohol solution system probably reached a state of equilibrium concentration with the water from the hydrogel and inside the hydrogel itself, causing the alcohol to penetrate into the loose spaces of the hydrogel itself and hence the measurement of the increase in water absorption. Nevertheless, this is a good phenomenon that shows the possibility of migration through and inside the hydrogel of liquids containing hydroxyl groups, and therefore of potential drugs that are soluble in these media.

Comparing the water absorption results for hydrogel samples with ginger (EEI) and hydrogel samples without EEI, with particular reference to the 4%Alg 12%PEGDA hydrogel, a much smaller increase in water absorption can be seen for samples with ginger extract added. These data are shown in Figure 3E,F. As already mentioned, the water absorption values for the sample with 22% EEI content reached the highest ESD value of about 0.042, and for the sample with 12% EEI content about 0.048. This is a low result, especially considering the maximum ESD value for the hydrogel sample with the same amounts of Alg and PEGDA in its composition, which is 1.07. Ginger present in the system at low concentrations can cause cross-linking of the system, thickening the spaces between the polymer chains, which in turn makes it difficult for them to bind with water, affecting the water absorption of the hydrogel.

Figure S2 (Supplementary Materials) shows the DSC curves. For each hydrogel, two peaks are visible, one of melting and evaporation, and the other of evaporation only. The endothermic peak around 0–10 °C is split into two smaller peaks. This is related to the melting and evaporation of differently bound forms of water. The first one is caused by the presence of bound water in the hydrogel sample, which freezes, while the second one is attributed to free and evaporating water, including that which has melted from the solid state [9]. For the hydrogel samples containing ginger extract, an increase in the intensity of the free water peak is visible—this peak lasts longer at temperature. This suggests that the share of loosely bound so-called “free” water in the hydrogel samples was higher than in the other hydrogels. Analyzing the data contained in Table 1 for the 4%Alg and 4%Alg 12%PEGDA 22%EEI hydrogels, the melting process starts at the same temperature of 3 °C and ends at 15 °C, but the first one reaches the highest peak value at 7 °C and the second at 10 °C.

The evaporation peak, similarly to the melting peak, is divided into two smaller peaks. The first one is associated with the evaporation of free water, which occurs at a temperature of approx. 100 °C. The second one is associated with the evaporation of water bound by stronger secondary bonds. The addition of PEGDA and ginger extract causes the evaporation peak to broaden and shrink in relation to the reference sample 4%Alg. Their expansion indicates the increased difficulty of water evaporation, caused by the high energy of bonds between polymer chains and water molecules, which requires a higher temperature to evaporate it (release it from the densely cross-linked polymer matrix). The highest value of the evaporation process end temperature, equal to 134 °C, is characteristic of the 4%Alg 12%PEGDA 12%EEI hydrogel. Additionally, it is also characterized by the lowest value of the temperature at which the evaporation process begins, equal to 58 °C. These results are shown in Table 2. This suggests that it is characterized by the highest obtained hydrophilicity, which makes it the best candidate for use as a dressing, taking into account this property. The extended evaporation peak of the hydrogel with a 4% alginate content indicates a high content of free water and weakly bound water in its structure.

The highest heat of transformation, i.e., the amount of energy required for the phase transition in a given hydrogel, for the melting and evaporation peaks, which have been listed in Table 2 and Table 3, is shown by the reference sample without the addition of PEGDA and EEI. This may indicate the amount of water absorbed by the 4%Alg hydrogel structure, due to its ability to move the main polymer chain relative to itself and the low degree of cross-linking of the structure.

The 4%Alg 12%PEGDA 12%EEI sample is characterized by the highest heat of transformation of all hydrogels containing additives in their composition. This value is similar to that of the other modified hydrogels. The EEI content of 22% does not increase the energy required to detach water molecules from the hydrogel structure, which may indicate that the ginger extract itself does not participate or participates only to a small extent in the formation of the three-dimensional network of the forming (cross-linking) hydrogel.

The content of free or weakly bound water was calculated using the values of endothermic changes of melting water obtained from the DSC test, using the following formula, and the results are presented in Figure S3 (Supplementary Materials) [9]:

$$W_{f}=\frac{∆H_{endo}}{∆H_{W}}\cdot 100\%$$

*W_f_*—percentage of unbound or weakly bound water in the hydrogel [%],

Δ*H_endo_*—heat of endothermic transformation of water in the hydrogel [J/g],

Δ*H_W_*—heat of fusion of pure water [J/g].

The equilibrium water content in the hydrogel (*W*_∞_) was calculated using the formula [9]:

$$W_{\infty }=\frac{{ESD}_{Max}}{{ESD}_{Max}+1}\cdot 100\%$$

*W*_∞_—equilibrium water content in the hydrogel [%],

*ESD_Max_*—maximum ESD value.

**Figure 4:**
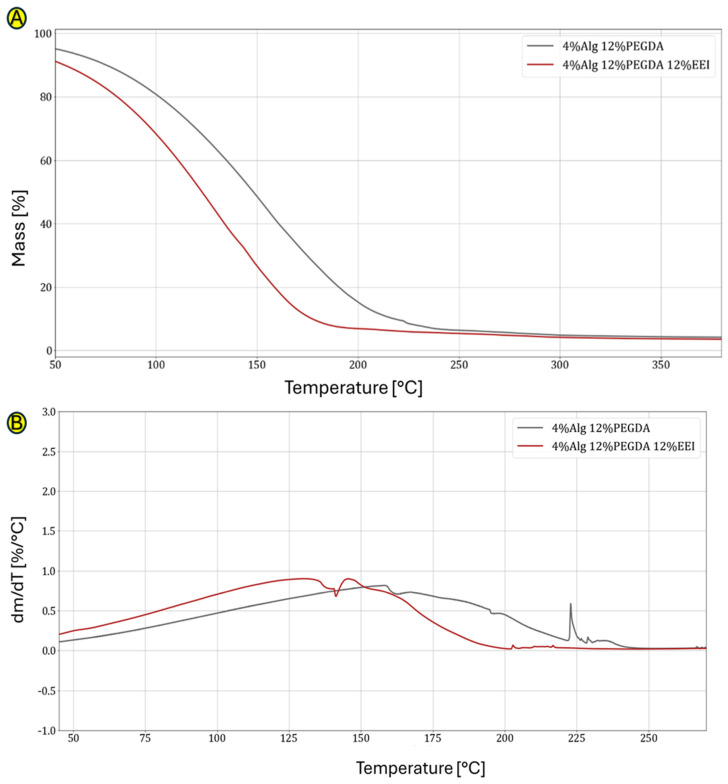
(**A**,**B**) Graphs showing comparison of TG curves for hydrogel without and with EEI addition (**A**) and comparison of DTG curves for hydrogel without and with EEI addition (**B**).

The data in Figure 4A confirm the previously obtained results. It was noticed that the value of the equilibrium water, i.e., water resulting from water absorption, is high for the 4%Alg 12%PEGDA hydrogel sample, which was characterized by the highest water absorption values in the water absorption test. However, it is low for the hydrogels with the addition of EEI, whose water absorption values were low.

Figure 4B compares the TG curves with and without the addition of ginger extract. The 12% content of ginger modifier affects the faster mass loss, which is mainly caused by water evaporation. This probably indicates the unstable connection of water molecules with the polymer chains from which this hydrogel is built. The difference between the hydrogel with the addition of EEI and without it is large. The loss of 50% of the mass of the first one is observed at a temperature of 126 °C, and the second at 150 °C (Table 4).

The DTG analysis shown in Figure 4B shows when the process of mass loss begins for EEI-modified and PEGDA-cross-linked samples and with what intensity these occur. As in the case of unmodified hydrogels, in this case, the greater the DTG peak flattening, the slower the hydrogel sample loses water. Due to the successive evaporation of different types of bound water in DTG, we observe one main flattened peak, indirectly defining the nature of the obtained spatial structure of the obtained and tested hydrogel materials. The addition of EEI causes the water in this type of hydrogel to evaporate much faster (at a lower temperature). This means that the presence of EEI affects the quality and density of hydrogel cross-linking. The DTG graphs show the beginnings of degradation of the main polymer network (approx. 225 °C).

The pH test (Table 5) showed that the addition of ginger extract and PEGDA caused a decrease in the pH value compared to the reference sample with 4% sodium alginate content. This difference after 24 h was 0.20 for the sample without EEI, 0.65 for the sample with 12% EEI and 0.69 for the sample with 24% EEI. Healthy skin has an acidic pH, while in the case of acute or chronic wounds, the pH increases. Therefore, it is desirable for the material supporting wound healing to affect the regulation (decrease) of the wound pH value. It was observed that the pH values in each of the measurement hours were the lowest for the sample with 22% EEI content. With the increase in the concentration of the ginger extract addition, the acidity of the solution increases.

During the ionic conductivity test, an increase in the value of this parameter was observed over time for all of the hydrogel samples tested (Table 6). This indicates the transfer of ions from the material to the solution and their increased mobility in the solution. The 4%Alg 12%PEGDA 12%EEI hydrogel was characterized by the highest increase in this parameter. This result is positive and suggests that this hydrogel releases an active substance that could be enclosed in the hydrogel and that it is permeable (diffusion) in its entire volume.

**Figure 5:**
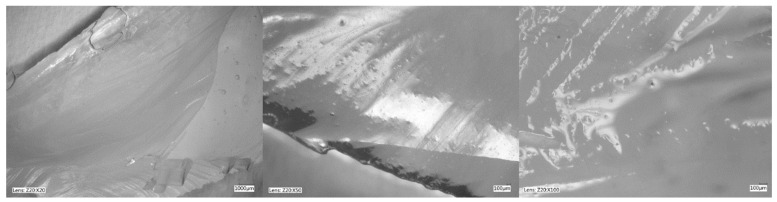
Hydrogel with 4%Alg. Photo from an optical microscope.

**Figure 6:**
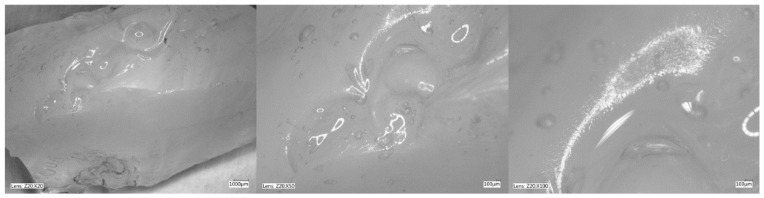
Hydrogel with 4%Alg 12%PEGDA. Photo from an optical microscope.

**Figure 7:**
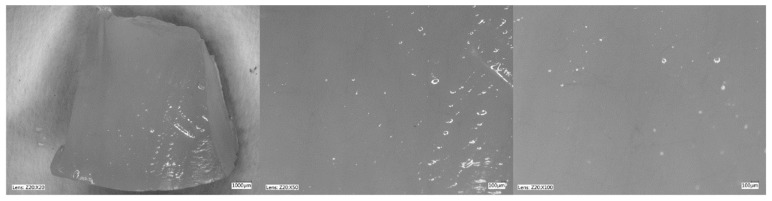
Hydrogel with 4%Alg 12%PEGDA 12%EEI. Photo from an optical microscope.

**Figure 8:**
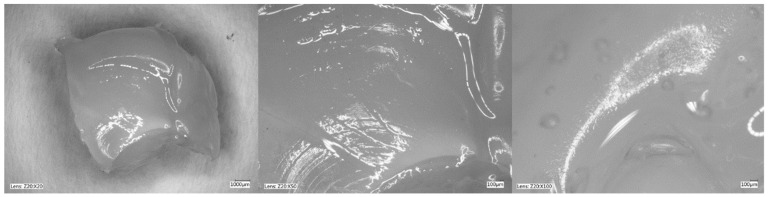
Hydrogel with 4%Alg 12%PEGDA 22%EEI. Photo from an optical microscope.

Figure 5, Figure 6, Figure 7 and Figure 8 show photos taken with a light microscope. The structure of the hydrogels obtained is homogeneous and smooth. The blisters visible in the photos were created as a result of mechanical mixing of the suspension before the cross-linking process. The structure of an ideal hydrogel dressing should be characterized by appropriate porosity. A porous scaffold promotes skin cell proliferation, ensures gas transmission and nutrient diffusion. Therefore, further work should focus on finding a suitable method to obtain a porous hydrogel with an appropriate pore size.

## 3. Discussion

Due to the very rapid development in the biomaterials and tissue engineering market, some innovative dressings enriched with natural bioactive substances have found wide application in the biomedical field, especially in wound care. Ismail et al. (2022) produced hydrogel dressings based on chitosan with the addition of ginger, curcumin and cinnamon [10]. Studies have shown that the chitosan/ginger dressing had the best mechanical properties compared to other samples and high antimicrobial activity against Gram-positive micro-organisms [10].

The authors of this manuscript proposed the design of hydrogel dressings based on sodium alginate and polyethylene glycol diacrylate with the addition of ethylene ginger extract as a bioactive molecule of natural origin. The functionalization of hydrogels with the addition of ginger essential oil allows the use of the plant’s antimicrobial properties to enhance the antimicrobial properties of wound dressings.

Hydrogels can effectively accelerate wound healing due to their many properties. One of the most important properties of hydrogels, in the context of assisting the management of skin wounds, is the ability of the dressing to absorb wound exudate and water, which directly reflects the ability to moisturize [11]. Additionally, the moist environment of the dressing allows the migration of epithelial cells and leukocytes and the distribution of growth factors to the wound bed, facilitating the healing process [12]. It has been observed that the addition of PEGDA can have both positive and negative effects on water absorption values. In the tests conducted, the greatest increase in water absorption was noted for the hydrogel sample with 4%Alg content/12%PEGDA content. Comparing the maximum ESD values of this sample and the reference sample (4%Alg), this value increased by 0.686.

Hydrogel dressings should have sufficient adhesion and appropriate mechanical properties to adhere and completely seal wounds under moist and dynamic conditions [13]. The 4%Alg/12%PEGDA hydrogel was characterized not only by the best water absorption values, but also by the slowest weight loss as a function of temperature, which was recorded during the TG tests. This indicates the high binding of its polymer chains to water molecules. Additionally, the 4%Alg/12%PEGDA hydrogel was also characterized by a relatively high tan(delta) value, and thus had the ability to dissipate stresses in its structure, a feature that can be correlated with the presence of a liquid phase and, in the case of a hydrogel, with a supporting phase. On the other hand, the studies by Ismail et al. (2022) showed that among hydrogel dressings based on chitosan with the addition of ginger, curcumin or cinnamon, it was the chitosan/ginger dressing that had the best tensile strength and elongation [10].

The hydrogels were modified by adding 12% and 22% ginger extract. It was found that the addition of the ginger modifier had a negative effect on the water absorption values. This may be due to additional cross-linking of the system, which is a process caused by the presence of EEI, which prevents the polymer chains from combining with water. A theoretical solution could be to obtain hydrogels with a higher EEI content. However, when analyzing the results, there is a decrease in the water absorption parameter is as the amount of EEI increases. This relationship may be due to an excess of modifiers in the composite. On the other hand, the results of the studies by Zhang et al., (2015) and Jun Yang et al. (2006) suggest that due to its natural composition, ginger may be beneficial for the preparation of hydrogels with high water absorption capacity [14,15]. It should be noted that despite the low water absorption values for samples containing ginger extract, an increase in this parameter over time is observed in the presented studies, which is a positive result.

The addition of PEGDA and EEI affects the broadening of the evaporation peak (DSC curve), indicating increased difficulty in evaporating water from hydrogels. This is probably related to the presence of more bound water in their structure, which freezes, than in other types of hydrogels. This may be indicative of their greater hydrophilicity, which is important for keeping the dressing moist for as long as possible and for maintaining a moist environment to promote faster wound healing. The analysis shows that the 4%Alg 12%PEGDA 12%EEI hydrogel is probably the most appropriate for this. The studies conducted indicate the importance of water, which can take on various functions, from loose to increasingly strongly bound to the polymer network, and which ultimately plays a key role in giving hydrogels not only mechanical but also functional properties.

The wound environment is characterized by an elevated pH, which is due to, among others, the presence of bacteria, while a lower pH reduces the bactericidal activity of the dressing. In addition, Sim et al. (2022) demonstrate the importance of maintaining an acidic wound microenvironment, which could be a potential therapeutic strategy for wound management [16]. Therefore, the ideal dressing should lower the pH value at the site of injury. The studies conducted have shown that both the addition of PEGDA and EEI increased acidity. On the other hand, Khan et al., (2020) in their studies, observed the stability of the pH parameter in gelatin/poly(vinyl alcohol) hydrogel films with the addition of ginger extract. As indicated by the authors, this could be due to the fact that the essential oil present in ginger is a natural preservative that prevents degradation [17,18].

The addition of PEGDA and EEI affects the broadening of the evaporation peak (DSC curve), indicating increased difficulty in evaporating water from hydrogels. This is probably related to the presence of more bound water in their structure, which freezes, than in other types of hydrogels. This may indicate their greater hydrophilicity, which is important for retaining moisture for as long as possible and for maintaining a moist environment to promote faster wound healing. The analysis shows that the 4%Alg 12%PEGDA 12%EEI hydrogel is probably the most appropriate for this. The studies carried out indicate the importance of water—which can take on various functions, from loose to increasingly strongly bound to the polymer network—in playing a key role in ultimately giving hydrogels not only mechanical but also functional properties.

Advanced wound dressings have emerged as promising tools in expediting the healing process. The wound environment is characterized by an elevated pH, which is evidenced by, among others, the presence of bacteria, while a lower pH affects the bactericidal activity of the dressing. Therefore, the ideal dressing should lower the pH value at the site of injury. The studies conducted have shown that both the addition of PEGDA and EEI increase acidity. On the other hand, Khan et al., (2020) in their studies, observed the stability of the pH parameter in gelatin/poly(vinyl alcohol) hydrogel films with the addition of ginger extract [18]. As the authors indicate, this could be due to the fact that the essential oil present in ginger is a natural preservative that prevents degradation [17,18]. Therefore, 4%Alg 12%PEGDA 12%EEI hydrogel can be used as a high-performance wound dressing, but further research is required.

## 4. Materials and Methods

**Scheme 1:**
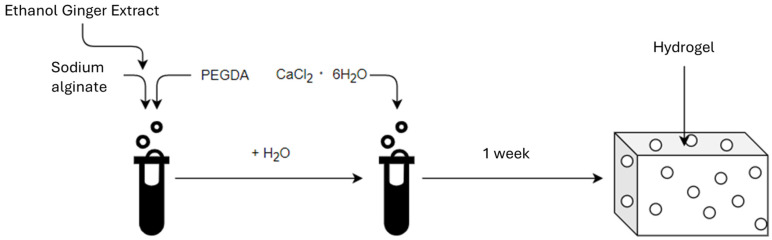
The scheme for obtaining the material.

The first stage of the research was to develop a hydrogel material (Scheme 1). Powdered sodium alginate (Alg), (ACROS Organic, Thermo Fisher Scientific, Geel, Belgium) and liquid polyethylene glycol diacrylate (PEGDA Mn = 700), (Sigma-Aldrich, Burlington, MA, USA) were used to produce the hydrogel matrix. Hydrated calcium chloride (6 H_2_O groups) CZDA (POCH Avantor Performance Materials Poland S.A., Gliwice, Poland) was used to cross-link the hydrogels. The ginger ethanol modifier was obtained using ginger powder (Kamis, Croton Falls, NY, USA) and anhydrous ethyl alcohol 99,8% CZDA (POCH Avantor Performance Materials Poland S.A., Gliwice, Poland).

**Figure 9:**
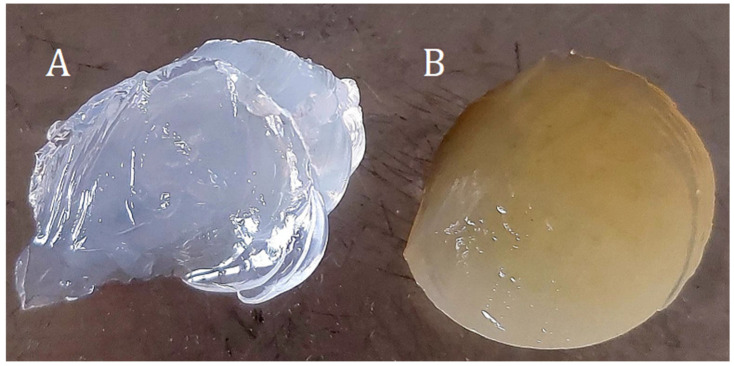
Hydrogel samples (**A**)—4%Alg 12%PEGDA, (**B**)—4%Alg 12%PEGDA 12%EEI.

*a.*  *Method of producing hydrogels based on alginate and polydiacrylate*
  Hydrogel systems based on sodium alginate (Alg) with a 7%, 9% and 12% addition of poly(ethylene glycol) diacrylate (PEGDA) were prepared at 4%, 3% and 2%. Nine samples were obtained, the percentage compositions of which are listed in Table 7. The cross-linking process lasted 7 days. Additionally, three reference samples were prepared without the addition of PEGDA (Table 8).
*b.*  *Preparation of hydrogels with ginger modifier.*
  Two samples were prepared with ginger modifier (EEI) at 12% and 22% EEI content (Table 9, Figure 9). The cross-linking process lasted 7 days.
*c.*  *Analytical methods of obtained hydrogels.*
  In order to determine the properties of the developed dressing, thermogravimetric (TG) examination, differential scanning calorimetry (DSC), dynamic mechanical analysis (DMA), water absorption tests and microscopic analyses were performed.
  i. The TG test was performed using a TG 550 Discovery thermogravimetric analyzer from TA Instruments. Samples weighing approximately 30 mg were heated in platinum crucibles to 500 °C at a rate of 10 °C/1 min in a nitrogen flow of 25 mL/1 min.
  ii. Differential scanning calorimetry (DSC) was performed to determine the type of water present in the hydrogel system. The choice of this method enabled the analysis of the melting and evaporation processes of the obtained hydrogels. A Mettler Toledo DSC differential scanning calorimeter was used. Samples weighing approximately 5 mg were prepared and subjected to temperatures ranging from −30 °C to 300 °C and a heating rate of 10 °C/min. A nitrogen atmosphere was set at a flow rate of 20 mL/min.
  iii. The DMA test was performed using a TA Instruments DMA 850 dynamic mechanical analyzer. Hydrogel samples were subjected to a constant temperature of 37 °C and a constant amplitude of 40 μm. The parameter that changed was the frequency, which oscillated between 1 Hz and 50 Hz, increasing every 5 Hz in each cycle.
  iv. Microscopic observations were carried out using a Keyence VHX-5000 digital microscope with a universal lens that allowed for image magnification from 20 to 200 times. The study allowed for the assessment of the microstructure of the obtained composite hydrogels and for examining their quality.
  v. The water absorption test was aimed at examining the amount of water absorbed and lost by the hydrogel. For this purpose, samples of cross-linked hydrogels were prepared (Figure S1), which were weighed (*W_x_*), then placed in separate containers and filled with 25 mL of deionized water and ethylene alcohol 99.8% CZDA.
  vi. The mass of the samples (*W_h_*) was measured after they had been filtered on filter paper at specific time intervals.
  vii. The equilibrium swelling degree (ESD) was calculated using the formula [18]:
    $$\mathrm{ESD}=\frac{W_{h}-W_{x}}{W_{x}}$$
    where
    ESD—water absorption value,
    *W_h_*—mass of the incubated sample [g],
    *W_x_*—mass of the dry sample [g].
  viii. pH and ionic conductivity tests were performed for the following hydrogels: 4%Alg, 4%Alg 12%PEGDA, 4%Alg 12%PEGDA 12%EEI and 4%Alg 12%PEGDA 22%EEI. Samples weighing from 1 g to 2 g were prepared (Table 10 and Table 11). Samples for pH testing were flooded with 20 mL of deionized water, while samples for ionic conductivity testing were flooded with 20 mL of Ringer’s solution. The test was performed by measuring the solutions before introducing the hydrogel samples into the solution, after 1 h, 2 h and 24 h of the samples’ presence in the solution. The pH test was performed using a pH meter. The ionic conductivity test was performed using a conductometer with a glass electrode.

## 5. Conclusions

The paper examines aspects of the mechanical properties of hydrogels, including conductivity, evaporation energy and thermal stability. Based on the conducted studies and obtained results, precise data were presented on the structure of the obtained hydrogels, and recipes for the synthesis of hydrogels intended for modern dressings were evaluated. The addition of ginger is innovative, because it itself contains biologically active compounds with properties such as anti-inflammatory, antibacterial and antiviral action. The addition of ginger to dressings is an extremely interesting and natural alternative to synthetic pharmacological preparations. However, it has an impact on the structure of the hydrogel, which was presented in detail in the paper. The studies conducted on ginger as an additive for hydrogel dressings do not exclude it as an additive, but they do note that ginger can slightly change the specificity of the material itself. Appropriate proportions and precise determination of the recipe for the synthesis of hydrogel with ginger should make such a composition possible to use as a modern hydrogel dressing for difficult-to-heal wounds.

## Figures and Tables

**Table 1:** The temperatures corresponding to the different degrees of mass loss for the hydrogels obtained without the addition of EEI.

Nr.	Sample	*T*_1%_ [°C]	*T*_3%_ [°C]	*T*_5%_ [°C]	*T*_10%_ [°C]	*T*_50%_ [°C]
1.	2%Alg 7%PEGDA	48	61	71	88	150
2.	3%Alg 7%PEGDA	42	52	59	72	123
3.	4%Alg 7%PEGDA	47	61	70	87	153
4.	2%Alg 9%PEGDA	47	59	67	83	145
5.	3%Alg 9%PEGDA	41	49	56	68	115
6.	4%Alg 9%PEGDA	47	57	65	80	141
7.	2%Alg 12%PEGDA	44	54	61	76	132
8.	3%Alg 12%PEGDA	42	52	60	74	130
9.	4%Alg 12%PEGDA	46	59	69	85	150

**Table 2:** Values of temperature, heat of transformation and enthalpy for the melting peak.

Nr.	Sample	*T_initial_* [°C]	*T_final_* [°C]	*T_peak_* [°C]
	4%Alg	3	15	7
1.	4%Alg 12%PEGDA	4	12	8
2.	4%Alg 12%PEGDA 12%EEI	1	14	11
3.	4%Alg 12%PEGDA 22%EEI	3	15	10

**Table 3:** Values of temperatures, heat of transformation and enthalpy for the evaporation peak.

Nr.	Sample	*T_initial_* [°C]	*T_final_* [°C]	*T_peak_* [°C]
	4%Alg	90	114	107
1.	4%Alg 12%PEGDA	89	112	105
2.	4%Alg 12%PEGDA 12%EEI	58	134	118
3.	4%Alg 12%PEGDA 22%EEI	73	98	93

**Table 4:** The temperatures corresponding to different degrees of mass loss for the hydrogels obtained without and with EEI addition.

Nr.	Sample	*T*_1%_ [°C]	*T*_3%_ [°C]	*T*_5%_ [°C]	*T*_10%_ [°C]	*T*_50%_ [°C]
1.	4%Alg 12%PEGDA	46	59	69	85	150
2.	4%Alg 12%PEGDA 12%EEI	42	50	57	71	126

**Table 5:** The pH test.

Sample	Quantity of Deionized Water [mL]	Sample Weight [g]	pH of Distilled Water	Measurement [pH]		
				After 1 h	After 2 h	After 24 h
4%Alg	20	1.207	5	5.52	6.12	6.20
4%Alg 12%PEGDA	20	1.721	5	5.38	5.94	6.00
4%Alg 12%PEGDA 12%EEI	20	2.175	5	5.72	5.54	5.55
4%Alg 12%PEGDA 22%EEI	20	1.189	5	5.35	5.43	5.51

**Table 6:** Ionic conductivity test.

Sample	Amount of Ringer’s Fluid [mL]	Sample Weight [g]	Ionic Conductivity of Ringer’s Solution	Measurement [μS]		
				After 1 h	After 2 h	After 24 h
4%Alg	20	2.441	40.1	40.80	42.50	41.30
4%Alg 12%PEGDA	20	1.356	40.1	40.20	43.20	43.20
4%Alg 12%PEGDA 12%EEI	20	1.943	40.1	43.30	40.70	44.90
4%Alg 12%PEGDA 22%EEI	20	1.279	40.1	42.70	40.70	44.10

**Table 7:** Contents of individual reagents in the obtained hydrogel samples.

Number	Sample
1.	2%Alg 7%PEGDA
2.	3%Alg 7%PEGDA
3.	4%Alg 7%PEGDA
4.	2%Alg 9%PEGDA
5.	3%Alg 9%PEGDA
6.	4%Alg 9%PEGDA
7.	2%Alg 12%PEGDA
8.	3%Alg 12%PEGDA
9.	4%Alg 12%PEGDA

**Table 8:** Prepared reference samples.

Number	Sample
1R	2%Alg
2R	3%Alg
3R	4%Alg

**Table 9:** Prepared samples with ethanolic ginger extract.

Number	Sample
1.	4%Alg 12%PEGDA 12%EEI
2.	4%Alg 12%PEGDA 22%EEI

**Table 10:** Masses of hydrogel samples for pH measurement.

Sample	Sample Weight [g]
4%Alg	1.207
4%Alg 12%PEGDA	1.721
4%Alg 12%PEGDA 12%EEI	2.175
4%Alg 12%PEGDA 22%EEI	1.189

**Table 11:** Masses of hydrogel samples for ionic conductivity measurement.

Sample	Sample Weight [g]
4%Alg	2.441
4%Alg 12%PEGDA	1.356
4%Alg 12%PEGDA 12%EEI	1.943
4%Alg 12%PEGDA 22%EEI	1.279

## Data Availability

Data are contained within the article.

## Supplementary Materials

The following supporting information can be downloaded at: https://www.mdpi.com/article/10.3390/ijms26073387/s1.
