# Supplementary material for: Application of Polysaccharides in Hydrogel Biomaterials
Source: Int J Mol Sci. 2025 Apr 4;26(7):3387. doi: 10.3390/ijms26073387 (PMC11989842; doi:10.3390/ijms26073387)
Supplement: Supplementary file 1 [file ijms-26-03387-s001.zip › ijms-3562706-supplementary.pdf]

Supplementary Information

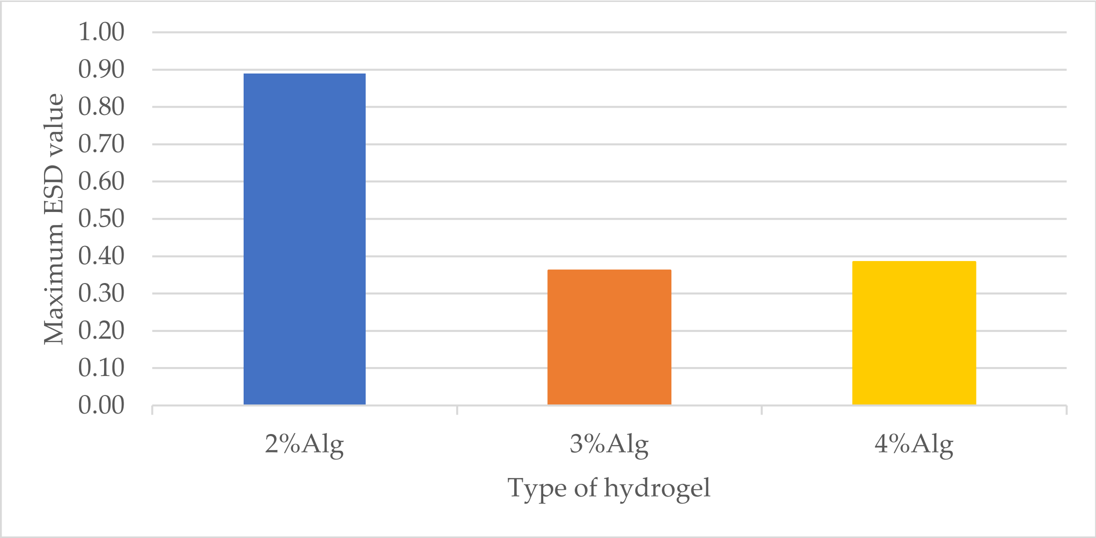

Figure S1a. Graph showing the maximum ESD coefficient values for hydrogel samples in deionized water

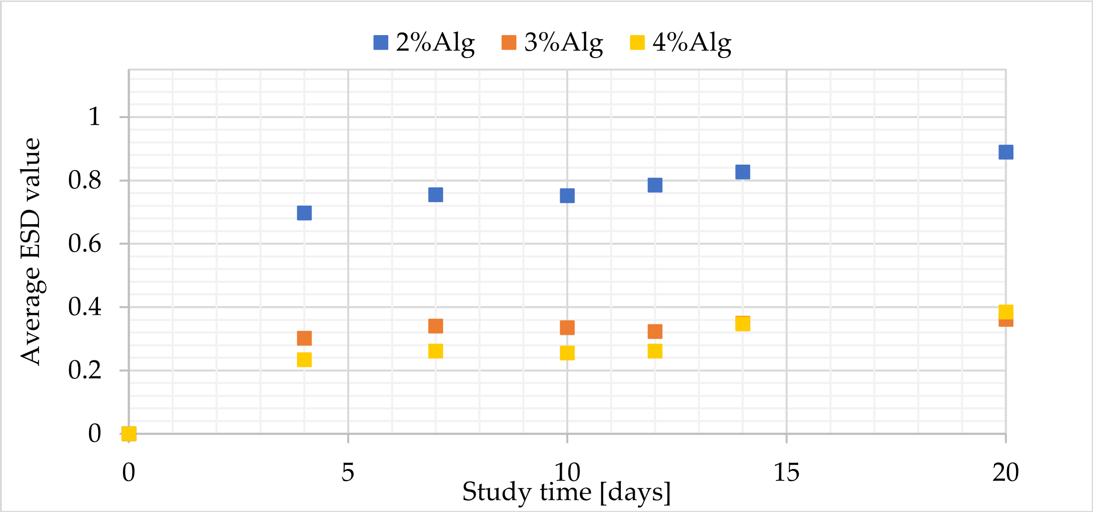

Figure S1b. Graph showing the maximum ESD coefficient values for hydrogel samples in deionized water

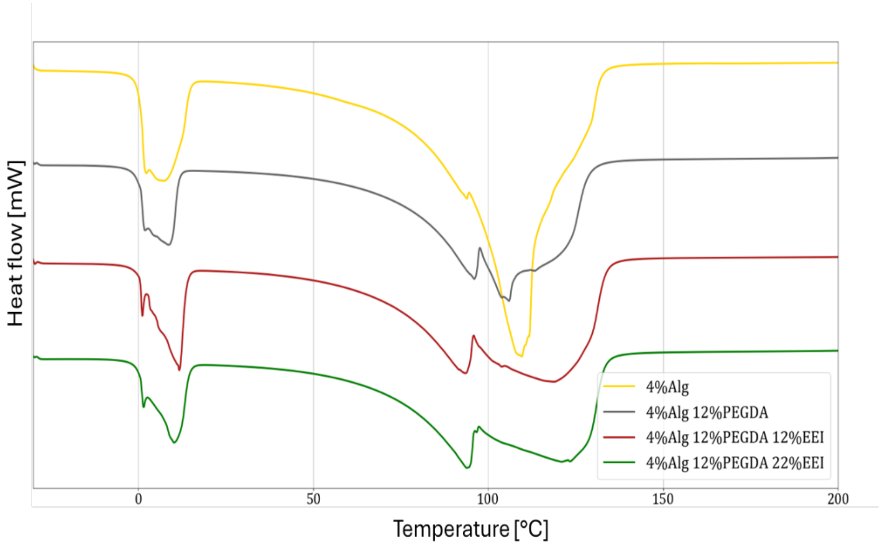

Figure S2. DSC curves for the obtained hydrogels without and with EEI addition.

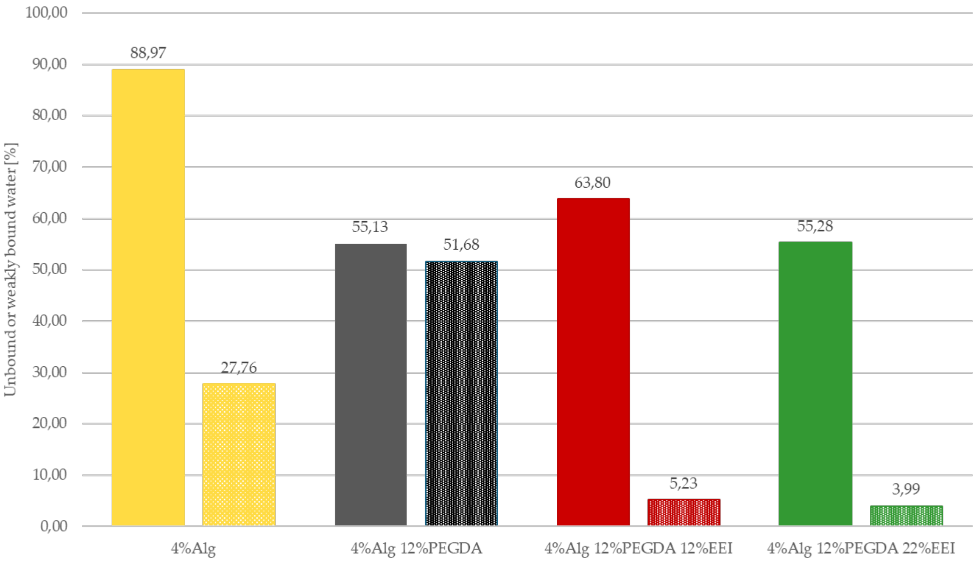

Figure S3. Comparison of the percentage of weakly bound or unbound water and water resulting from water absorption for selected hydrogels.
